# Treatment of Schatzker Type VI Tibia Fractures Using Circular External Fixation: State of the Art, Surgical Technique and Results

**DOI:** 10.3390/jcm13051249

**Published:** 2024-02-22

**Authors:** Javier Martínez Ros, Alonso Escudero Martínez, Miguel Martínez Ros, José Molina González, María Carrillo García, Juan Pedro García Paños, José Pablo Puertas García-Sandoval, César Salcedo Cánovas

**Affiliations:** 1 Servicio de Cirugía Ortopédica y Traumatología, Unidad de Patología Séptica Osteoarticular, Hospital Clínico Universitario Virgen de la Arrixaca, 30120 Murcia, Spain; jmolinagonz@yahoo.com (J.M.G.); cesalcan@yahoo.es (C.S.C.); 2 Servicio de Cirugía Ortopédica y Traumatología, Hospital Clínico Universitario Virgen de la Arrixaca, 30120 Murcia, Spain; alonso.m.e.1992@gmail.com (A.E.M.); mmr16@hotmail.com (M.M.R.); drjpgarciapanos@gmail.com (J.P.G.P.); jppuertasgsandoval@gmail.com (J.P.P.G.-S.); 3 Servicio de Radiodiagnóstico, Hospital Clínico Universitario Virgen de la Arrixaca, 30120 Murcia, Spain; mariacg7891@gmail.com

**Keywords:** Schatzker, tibia, fracture, external fixation, Truelok, knee, Ilizarov

## Abstract

Background: Schatzker type VI tibia fractures are usually associated with infection and surgical wound-related problems. Circular external fixation (CEF) has been shown to minimize such complications. Methods: We pose a retrospective study of patients with Schatzker type VI fractures treated with CEF. Results: Twenty-two (22) patients were included (11M/11F) with a mean age of 60.1 ± 14.9 years. According to the AO/OTA classification, two fractures (9.1%) were A2, three (13.6%) were A3, and seventeen (77.3%) were C3. Three (13.6%) of them were open. The tissue damage observed in the nineteen (86.4%) closed fractures was classified according to Tscherne (four grade I, twelve grade II, and three grade III). The mean ex-fix time was 24.1 ± 5.1 weeks. None of the patients experienced deep infections, nonunion, or malunion. The mean ROM was 111.4 ± 17.8 degrees. Although stability was achieved in all cases, 50% of them suffered osteoarthritic degeneration. Four knees required TKR at a mean of 8.77 ± 5.58 years from trauma. The mean HHS knee score was 84.2 ± 10.3 points (excellent in fifteen (68.2%) cases, good in four (18.2%), and acceptable in three (13.6%)). The mean Rasmussen radiological score was 13.3 ± 3.5 (excellent in three (13.6%) cases, good in fifteen (68.2%), and acceptable in four (18.2%)). The mean SF-12 score was 35.1 ± 10.4 points on the physical scale and 53.0 ± 10.6 points on the mental scale. Conclusions: CEF has shown itself to be a valid treatment for patients with Schatzker type VI fractures, particularly for those where the fracture is comminuted, severely displaced, open, or associated with severe soft tissue damage.

## 1. Introduction

Although Schatzker type VI proximal tibial fractures [1] account for less than 1% of all fractures [2,3,4], they are usually associated with a poor prognosis. Typically caused by high-energy trauma, these fractures tend to result in extensive bone comminution, significant soft tissue damage, neurological impairment, and/or compartment syndrome [2,5,6]. Choice of treatment is often dictated by host-related factors, the condition of the soft tissues, and the severity of the injury [7].

The purpose of the treatment of these fractures is the same as that for any other joint fracture, i.e., restoring the joint line and the alignment of the limb, limiting the morbidity of surrounding tissues, and preserving joint stability and function in an attempt to prevent post-traumatic osteoarthritis [5,6]. Against this background, careful management of the soft tissues is of particular importance given the limited soft tissue coverage in that area of the anatomy and the severe damage that such tissues are prone to.

For many years, open reduction by means of an extensive anterior incision allowed excellent visualization of the surgical field and a consequently accurate reduction but, more often than not, resulted in periosteal devascularization and additional soft tissue damage, which increased the risk of skin necrosis and/or infection [8,9,10]. It was not until the advent of low-profile anatomical locking plates that healing and infection rates improved through less invasive and more soft tissue-sparing approaches. That was the time when the concept of biological fixation was introduced and when authors became aware of the importance of bridging areas of severe comminution or, alternatively, performing an indirect reduction in the fracture [6,9]. However, internal fixation technologies and minimally invasive approaches have not been able to provide a lasting solution, particularly in cases of extensively comminuted fractures and severe soft tissue damage [5,9].

As a result, bridging external fixation has gained popularity as a local damage control strategy aimed at decreasing inflammation, gaining access to soft tissues to monitor their condition, controlling pressure in the affected compartments, and mobilizing patients ahead of a potential (definitive) internal fixation treatment [5,6,11,12]. Some authors are now using external fixation—on its own or in combination with minimal internal fixation—as a definitive treatment for severely comminuted fractures [4,13,14,15,16,17,18,19]. In particular, circular external fixation has been shown to minimize damage to the soft tissue envelope, allow correction of the deformity at various levels, permit secondary corrections, provide early knee mobilization, and facilitate bridging of the joint in patients with concomitant ligament injuries [5,8].

Evaluating the progression of treatment and the results obtained in patients with Schatzker type VI fractures is particularly difficult given the heterogeneity of cases. For one thing, most cases belong to one of two groups: young patients with high-energy fractures and elderly patients with low-energy injuries [20]. Secondly, the most commonly used scales (Schatzker [1] and AO/OTA [19]) do not take into account such prognosis-modifying factors as the degree of cartilage and ligament involvement or the presence of related conditions. Finally, the condition of the soft tissues is so variable that each case could be treated on its own merits, which stands in the way of making homogeneous comparisons.

Thus, and regardless of the validity of other types of treatment, circular external fixation seems to have a space in the management of this type of trauma, and our hypothesis is that its clinical effectiveness as a definitive treatment is equivalent to other interventions. The purpose of this study was to determine the clinical effectiveness of circular external fixation as definitive treatment in these patients and to compare the series results with those of other series in the literature.

## 2. Materials and Methods

A retrospective review was carried out of our hospital’s database to identify all the patients who had been treated for a Schatzker type VI proximal tibia fracture with a circular external fixator from 1 January 2002 to 1 January 2021. We prescribe circular external fixation to all patients with Schatzker type VI fractures who present with significant comminution or fragment displacement, an open fracture, or severe soft tissue damage. Circular fixators are also applied to patients subjected to a fasciotomy due to compartment syndrome and to those with severe comorbidities. Different methods may be employed in treating other patients.

To be included in the study, patients were required to be over 12 years of age and had to have been treated by the hospital’s adult patient unit for an open or closed Schatzker type VI proximal tibia fracture. Patients were required to have been followed up for a minimum of 12 months after completion of treatment.

Patients followed up for less than 12 months or with incomplete postoperative follow-up were excluded from the analysis.

The study was approved by the Ethics Committee of the Virgen de la Arrixaca University Clinical Hospital on 6 January 2022.

### 2.1. Surgical Technique

**Figure 1:**
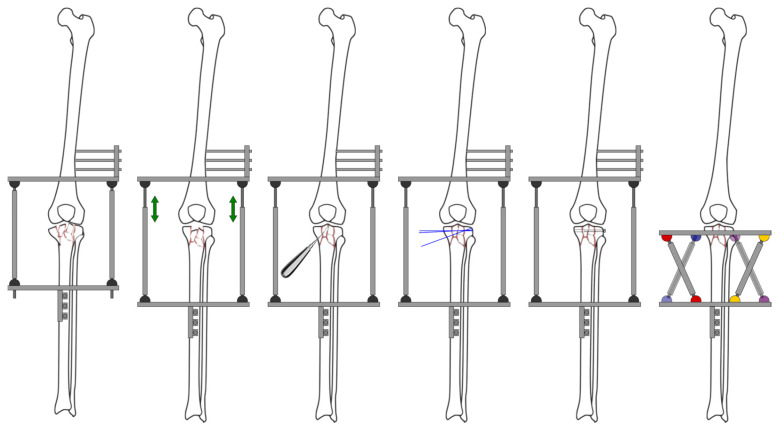
Schematic representation of the fracture reduction and fixation technique. From left to right: Attachment of the frame to both the femur and the tibia; ligamentotaxis through distraction (green arrows) of the external fixation frame; reduction in the central fragments; temporary articular fragment fixation using Kirschner wires; articular line fixation using lag screws; circular fixator in place using fine wires to stabilize fracture fragments.

**Figure 2:**
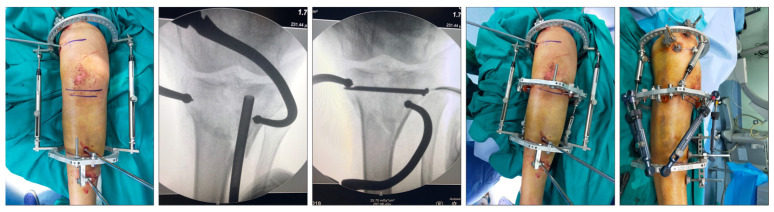
Fracture reduction and fixation in a real patient.

Figure 1 shows a schematic representation of the fracture reduction and fixation technique used in this series. Figure 2 shows a real case.

#### 2.1.1. Objectives

The purpose of the procedure was to achieve bone healing and restore as much of the patients’ previous range of motion and function as possible, minimizing the number of complications and the severity thereof. An effort was also made to restore the leg’s mechanical and anatomical axes, as well as the articular line, to minimize the risk of post-traumatic osteoarthritis.

#### 2.1.2. Imaging Tests

In addition to plain anteroposterior and lateral radiographs, a computerized axial tomography (CT) scan was performed with coronal, sagittal, and 3D reconstructions. Three-dimensional visualization of the fracture allows accurate identification of the fragments, facilitating restoration of the joint surface [5].

#### 2.1.3. Reduction and Fixation Strategy

Although ligamentotaxis usually helps restore the fragments’ natural position (something crucial to achieve good results), it may not always be entirely effective in addressing the central fragments of the joint, which are not connected with other tissues. In these cases, the reduction should be carried out using elevators, which must be inserted into distal metaphyseal windows, and be accompanied by minimal internal fixation. Under no circumstances should the metaphyseal–diaphyseal segment be fixed, as this would interfere with the reduction achieved by the external fixator and its subsequent dynamization. Bone grafting could in some cases enhance stability.

The external fixator plays a triple role: fixing the joint fragment by means of pins, assisting in the reduction in the fracture, and preserving limb length. The most recent patients in this series were treated with a telescoping bar system equipped with a software that allowed the performance of secondary corrections as needed (TrueLok Hex, Version 2.2, Orthofix Srl, Bussolengo, Italy).

Application of the fixator started at the proximal segment, placing at least three olive pins at the level of the fibular head. Stability was maximized through a large crossing angle. Rings were placed in the distal region and were fixed by means of hydroxyapatite-coated screws (currently just one ring is enough thanks to the use of recently introduced hexapod fixators). The fixator’s telescoping bars allowed performance of additional manoeuvres to ensure perfect limb alignment. Finally, all the fixator’s mobile components were locked.

**Figure 3:**
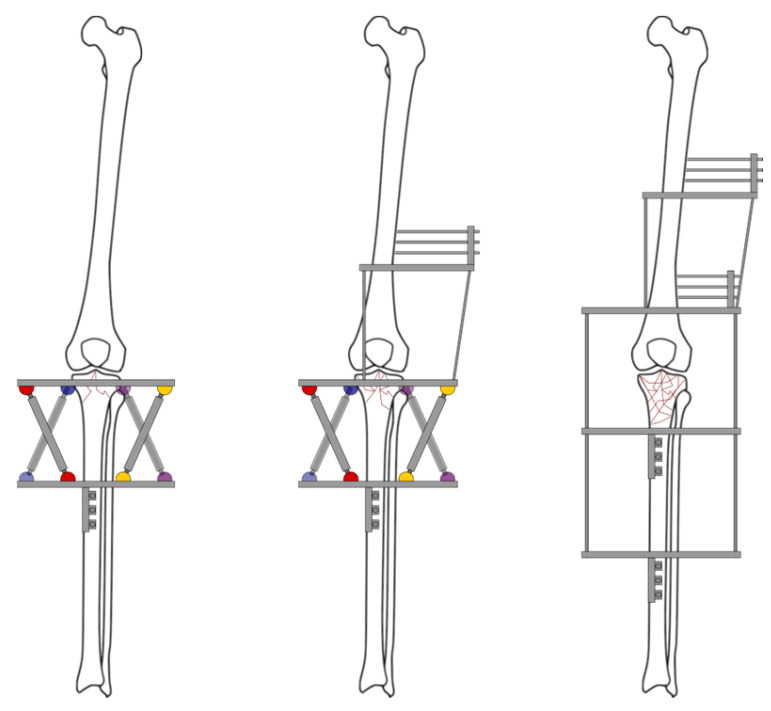
Possible external fixator configurations. From left to right: Non-bridging frame; joint-bridging frame; joint-bridging frames with no anchorage to the proximal tibia.

Following Catagni et al. [21], three possible external fixator configurations can be used (Figure 3):

- Non-bridging frames, which allow immediate joint motion: These are used when the joint is stable.
- Joint-bridging frames: Used in cases with marked joint instability and severe comminution.
- Joint-bridging frames with no anchorage to the tibia: Used when there is significant joint instability, severe comminution, and soft tissue compromise (either poor soft tissue quality or insufficient coverage).

#### 2.1.4. Rehabilitation

Patients must follow an appropriate rehabilitation protocol that allows progressive weightbearing and gradual resumption of everyday activities, promoting osteogenesis and reducing muscle atrophy and loss of range of motion. As regards weightbearing, two different situations can be identified:

- Non-bridging frames: Normally, 10 kg weightbearing is allowed during the first week, which is gradually increased until 30 kg is reached at the end of the first month. Thereafter, progressive increases are made until full weightbearing.
- Joint-bridging frames: Initial weightbearing may be 20 to 30 kg, which is progressively increased. The bridge is removed at the outpatient clinic between postoperative weeks 4 and 6.

### 2.2. Definition of Results

The patients’ anthropometric data was recorded, and fractures were classified according to the Schatzker [1] and AO/OTA scales [22]. Open fractures were also classified according to the Gustilo and Anderson grading system [23,24], and closed fractures were classified according to the Tscherne grading system [25]. The host’s status was staged using the Cierny–Mader classification [26]. An analysis was also conducted of surgical procedure-related data such as the number of days elapsed between trauma and surgery, the length of hospital stay, and the occurrence (or otherwise) of intraoperative complications. A record was kept of the time to external fixator removal and of the complications occurring during treatment.

The degree of function was determined by evaluating the stability and range of motion of the knee joint, checking for the appearance of post-traumatic osteoarthritis, and calculating the Hospital for Special Surgery (HHS) knee score [27]. Radiographs were assessed using Rasmussen’s radiological score [28], and quality of life was measured by means of the SF-12 physical and mental health summary scales [29].

### 2.3. Statistical Analysis

The data were summarized by means of arithmetical means and percentages. Variability was evaluated by standard deviation and comparisons between this series, and the mean values reported in the literature were determined using a one-sample Mann–Whitney U test. The sample size was too small to carry out statistical comparisons of qualitative variables. Statistical significance was set at a *p* value < 0.05. All calculations were made with the Stata v.14.0 statistical software (StataCorp, Lakeway Dr. College Station, College Station, TX, USA).

### 2.4. Comparison with Other Studies

A literature search was performed to determine whether the results obtained from this study were in line with those of similar publications. In eight of the studies analysed (three of them comparative) [4,13,14,15,16,17,18,30], circular external fixation was used as the only definitive treatment for Schatzker type VI fractures. Circular external fixators [8,21,31,32,33,34,35,36,37,38,39,40,41,42,43,44,45,46,47,48,49,50,51] were also used in another 22 of the studies reviewed to treat Schatzker type VI fractures, but these studies were excluded as they also included other types of fractures, which would invalidate any comparison.

## 3. Results

### 3.1. Results of This Series

The results of this study are summarized in Table 1. A total of 22 patients were treated for a Schatzker type VI fracture during the study period by circular external fixation. One of them died while participating in the study for reasons unrelated to their trauma. The mean patient age was 60.1 (±14.9) years, the sample being evenly distributed with regard to sex (11 males and 11 females). All fractures included in the study were Schatzker type VI fractures. Fractures were also graded on the AO/OTA classification, according to which two (9.1%) were type A2, three (13.6%) were type A3, and seventeen (77.3%) were type C3. Three (13.6%) of the fractures were open, one of them being graded as type IIIA and the other as type IIIB on the Gustilo and Anderson classification. The tissue damage observed in the 19 (86.4%) closed fractures was of grades I (four cases), II (twelve cases), and III (three cases) according to Tscherne’s classification.

The mean time to surgery was 10.4 (±5.7) days and the mean hospital stay was 17.6 (±9.7) days. Two patients required the use of bone grafting to fill the defect following reduction, and in another two the joint was bridged to protect the reconstruction and the ligaments. Close reduction was sufficient in all cases. None of the patients experienced deep infections, nonunion, or malunion. The mean time to external fixator removal was 24.1 (±5.1) weeks.

The mean range of motion was 111.4 (±17.8) degrees. All patients achieved full extension, except for one who maintained a 10 degree extension lag. Four of the patients were unable to achieve 90 degrees of flexion. All knees were stable, although 50% (11 cases) experienced some degree of osteoarthritic degeneration. Four patients required implantation of a total knee replacement at a mean of 8.77 (±5.58) years from trauma.

The mean HHS knee score was 84.2 (±10.3) points, with fifteen patients (68.2%) achieving an excellent result, four (18.2%) achieving a good result, and three (13.6%) achieving an acceptable result. The mean Rasmussen radiological score was 13.3 (±3.5) points, with three (13.6%) patients achieving an excellent result, fifteen (68.2%) achieving a good result, and four (18.2%) achieving an acceptable result.

The mean score on the SF-12 questionnaire was 35.1 (±10.4) points on the physical scale and 53.0 (±10.6) points on the mental health summary scale.

### 3.2. Comparison with the Results of Other Studies

The main results of the comparison carried out are shown in Table 2. The sample size of this study is in line with that of the eight studies analysed (22 vs. 26.1; *p* = 0.866), although the proportion of male subjects is lower in the present study (50% vs. 69.4%) and our mean age is significantly higher (60.1 vs. 45.7 years; *p* = 0.022). On the other hand, the percentage of open fractures in this series is smaller than that in the studies considered (13.6% vs. 39.5%). Only two of the studies analysed (El Barbary et al. [14] and Debnath et al. [16]) reported tissue damage following closed fractures, yet their incidence was lower than the 86.4% found in this series (40% and 70%, respectively).

The time to surgery in this series was similar to the mean value reported by other authors (10.4 vs. 9.2 days; *p* = 1). In contrast, the time to removal of the external fixator was longer—albeit not statistically significantly so—than that reported in the literature considered (24.1 vs. 17.4 weeks; *p* = 0.059). The mean follow-up of our subjects was also longer than that in other studies, yet the difference was not statistically significant (74.0 vs. 41.7; *p* = 0.058).

As regards the results obtained, the fact that all our patients achieved bone healing without complications is statistically consistent with the results reported in the literature, where 2.6% of subjects presented with nonunion and 7.8% experienced longer healing times. The percentage of patients with good or excellent radiographic results and with good or excellent clinical results was almost identical to that of the other publications analysed (77.3% vs. 77.8% and 86.4% vs. 80.3%, respectively). As far as complications were concerned, no statistically significant differences were found regarding the pin tract infection rate (18.2% vs. 26.7%), the deep infection rate (0% vs. 2.3%), or the percentage of subjects presenting with osteoarthritic symptoms (50% vs. 56.4%).

## 4. Discussion

According to the results obtained, which are in line with those published by other authors, circular external fixation could be considered a useful treatment for patients with Schatzker type VI fractures, especially in cases of severely comminuted or displaced fractures, open fractures, or fractures associated with severe soft tissue damage. Despite the difficulties inherent in those fractures, the treatment applied resulted in satisfactory clinical and functional outcomes, with all patients achieving full fracture healing and experiencing no deep infections.

Osteosynthesis is the art of striking a delicate balance between mechanical stability and preservation of the biological environment. Although minimally invasive internal fixation using stable-angle implants has provided excellent results in the management of proximal tibia fractures [9,30,47,49,51,53,54,55,56], their use may require extensive dissections and direct manipulation of the fragments, which nullifies most of the benefits of percutaneous surgery [5,9]. For that reason, even if internal fixation allows better visualization of the joint surface and makes it easier to recognize and repair associated meniscal and collateral ligament injuries [6], several authors [4,13,14,15,16,17,18,19] consider that external fixation (either on its own or in combination with minimal internal fixation) ought to be the method of choice for treating comminuted or severely displaced fractures, or those associated with severe tissue damage.

One of the first factors that stands out about this series is the elevated mean age of the subjects included (60.1 years, range: 35–89), who were nearly 15 years older than those in the other studies considered, although in none of them did the authors include an age-related exclusion criterion. It must be noted, however, that two of the studies [4,18] excluded pathological fractures, which may have resulted in the exclusion of fractures caused by osteoporosis. According to the literature, tibial plateau fractures tend to exhibit a bimodal distribution, being concentrated in young patients with high-energy trauma and in elderly patients experiencing low-energy falls from a height [20]. Although the size of this sample was too small to draw hard-and-fast epidemiological conclusions, our data did not disprove this finding. In fact, some authors consider that, given the aging of the population, this type of fracture could come to be classed under the osteoporotic fracture category in the future, which will undoubtedly have implications for the way they are prevented and treated [3].

Another aspect that sets our series apart from the others is the relatively low percentage of open fractures included (13.6% vs. 39.5%). We believe this could be attributed to the importance given in our department to the condition of soft tissues in patients with closed fractures. All our closed fractures (n = 19) presented with tissue damage (four grade I, twelve grade II, and three grade III according to the Tscherne classification [25]). Unfortunately, a comparison with the findings of other authors is not possible as only El Barbary et al. [14] conducted a systematic classification of soft tissue status in closed fractures, observing soft tissue damage in 85.7% of closed fractures (six grade I, nine grade II, and three grade III). For that reason, it must be borne in mind that, despite their widespread use, the Schatzker et al. [1] and the AO/OTA [22] classifications do not consider the fracture’s degree of displacement, the associated soft tissue damage, or the status of the blood supply in the area. We therefore consider it necessary to use those classification systems in combination with the Gustilo and Anderson system [23,24] (for open fractures) or Tscherne’s system [25] (for closed fractures). Otherwise, there is a risk of comparing fractures with widely different characteristics.

The mean time to surgery in this series was also similar to that of previous publications (10.4 vs. 9.2 days; *p* = 0.831). It is not possible, however, to determine whether there existed differences regarding the length of hospital stay between the studies as this endpoint is only analysed by Debnath et al. [16] and by Ali et al. [15] (37.8 and 8 days, respectively). Despite this, we believe that the length of hospital stay tends to be more heavily influenced by the patients’ associated conditions and by hospital protocols than by the circumstances surrounding the surgical procedure and the subsequent reconstruction.

The time to removal of the external fixator in our patients was found to be significantly longer than reported by the other studies considered (24.1 vs. 18.4 weeks; *p* = 0.019). Although the heterogeneity of Schatzker type VI fractures may explain the significant differences between this analysis and the other studies analysed regarding time to removal of the external fixator, an effort was made herein to find more specific causes for such differences. Firstly, it must be pointed out that, in this study, fracture healing was not established using strictly radiographic criteria but, rather, fractures were deemed to have healed when the patient had been bearing full weight with the fixator for some time. On ascertaining that the fracture had healed, the external fixator was removed. Secondly, patients in this study were not splinted on removal of the fixator. In contrast to this study, one of the series where the time to removal of the external fixator was shorter [15,16] included two cases of deformity resulting from early removal of the fixator assembly (Ali et al. [15]) and the other reported a 73% splinting rate following removal of the external fixator (Debnath et al. [16]). These authors also reported a case where healing took longer than six months, which they do not seem to have taken into consideration for making their time-to-removal calculations. Against this background, it would seem that our cautious stance combined with the inconsistent criteria used by other authors in their calculations could, to a certain extent, explain the differences observed regarding the time to removal of the external fixator. According to our current protocol, fixators are almost invariably removed at four months, with no healing problems being observed.

In this series, all fractures went on to heal without the need of additional surgeries. It must be said, however, that, in four patients (18.2%), the time to heal was between 6 and 9 months. Although these cases could technically be regarded as delayed healing cases, which is what we have done, it is also true that two of the patients were over 75 and another two had sustained severe multiple traumas, which made a long wait necessary before the external fixator could be removed. These results seem to be in line with the data published by other authors. In any case, the definition of delayed healing often proves problematic. It should be remembered that in our department we only consider that the fracture has healed when we believe it is safe to remove the external fixator, which may result in increased healing times as compared with other series.

As regards clinical results, the mean HSS knee score for this series’ patients was 84.2 (±10.3). This means that 86.4% of our patients obtained either excellent or good clinical results, slightly better than the results of published series (80.3%). The situation is similar regarding radiological results, where no differences were found between this series and the others (77.3% vs. 77.8%).

Quality of life in our patients was evaluated using the SF12 physical and mental health summary scales, which were interpreted in accordance with the specific norms developed for the Spanish population [57]. It was observed that, within their age group, our patients fell into percentiles 10–20 on the physical scale and 50–60 on the mental scale. These scores must be considered in the light of the significant physical impairment caused by Schatzker type VI fractures which, more often than not, are associated with multiple traumas.

One of the theoretical advantages of external fixation lies in the fact that it allows preservation of the soft tissue envelope around the fracture [4,13,14,15,16,17,18,19], which typically results in a lower incidence of deep infection. This was indeed observed both in the present series, where no cases of deep infection were found, and in the previous series analysed, where the mean incidence of deep infections was only 2.3%. On the other hand, a theoretical disadvantage of external fixators is the risk of pin tract infection. Thanks to the extensive experience accumulated by our hospital in the use of external fixation in both trauma and limb reconstruction cases [52], we managed to keep superficial infections at a rate of 18.2%, which is lower than the mean rate observed in the studies under consideration (26.7%).

Although the cartilage damage associated with Schatzker type VI fractures tends to impact patient outcomes, no classification system considers the damage sustained by the joint, the menisci, or the adjacent structures [6]. These injuries may, in the medium or long term, lead to osteoarthritic degeneration of the knee. In fact, 50% (11) of our patients developed some measure of osteoarthritis. This is in line with the findings of other studies, where the percentage of individuals with osteoarthritic symptoms is as high as 56.4%. In this series, although four patients eventually underwent a total knee arthroplasty, we were able to stave off surgery for a considerable period, with a mean time from trauma to prosthetic replacement of 8.77 years.

Although there is a considerable number of publications comparing the effectiveness of external vs. internal fixation in the treatment of proximal tibia fractures [8,32,43,44,45,46,47,48,49,50], only three of the studies analysed focus specifically on Schatzker type VI fractures [4,18,30]. However, none of these studies is randomized and, in all three cases, the authors state that patients treated with external fixation were those with severe tissue damage, open fractures, multiple traumas, or severe comorbidities. Even in these unfavourable conditions for external fixation, none of the studies demonstrated significant differences between both kinds of treatment. Ahearn et al. [4] concluded that, although the fixation method used is not the make-or-break factor for successful treatment, in some fractures it is more advisable to resort to external fixation. Bove et al. [18] claim that, given its low invasiveness, external fixation typically results in fewer complications in high-risk patients, which makes it the ideal treatment for high-energy fractures associated with soft tissue damage, open fractures, or multiple traumas. Finally, Berven et al. [30] consider that, given the similarity between the clinical outcomes obtained with the different techniques, the most important success factor is the experience of the hospital where the treatment is administered.

This study is not without limitations, the most obvious being the retrospective nature of this series. It should also be borne in mind that the nature of the pathology prevents a large sample size and that not all fractures are treated equally, as some include the bridging of the joint and a different rehabilitation protocol. However, our critical analysis of the existing literature, combined with our own experience, makes us categorically reject the use of internal fixation for some patient populations and fracture types. Although minimally invasive internal fixation has a clear role to play in the treatment of proximal tibia fractures, we believe that in comminuted or severely displaced open articular fractures, in fractures involving significant tissue damage, and in patients with multiple traumas or severe comorbidities, external fixation offers undeniable advantages including the prevention of potential complications.

## 5. Conclusions

Circular external fixation is a valid alternative for managing patients with Schatzker type VI fractures and it offers particular advantages in patients with soft tissue damage, open fractures, multiple traumas, and severe comorbidities.

## Figures and Tables

**Table 1:** Summary statistics from this sample.

Variables		N (%)	Mean	SD	Min	Max
	Age	-	60.09	14.88	35	89
Demographics	Sex (M/F)	11/11 (50%)	-	-	-	-
	Laterality (R/L)	11/11 (50%)	-	-	-	-
AO/OTA classification	41-A2	2 (9.1%)	-	-	-	-
	41-C2	3 (13.6%)	-	-	-	-
	41-C3	17 (77.3%)	-	-	-	-
Open or closed fracture	Open fracture	3 (13.6%)	-	-	-	-
	Closed fracture	19 (86.4%)	-	-	-	-
Open fractures (G&A)	IIIA	1 (33.3%)	-	-	-	-
	IIIB	2 (66.7%)	-	-	-	-
Closed fractures (Tscherne)	Grade I	4 (21.1%)	-	-	-	-
	Grade II	12 (63.2%)	-	-	-	-
	Grade III	3 (15.8%)	-	-	-	-
Hospital	Time to surgery (days)	-	10.41	5.71	2	23
	Hospital stay (days)	-	17.59	9.62	5	38
	Bone graft	2 (9.1%)	-	-	-	-
	Knee span	2 (9.1%)	-	-	-	-
Results	Follow up (months)	-	74.00	62.31	21	232
	ExFix time (weeks)	-	24.08	5.09	16.43	36.71
	ROM (degrees)	-	111.36	17.81	70	130
	Unstable knee	0 (0%)	-	-	-	-
	Non-union	0 (0%)	-	-	-	-
	Malunion	0 (0%)	-	-	-	-
	Osteoarthritis	11 (50%)	-	-	-	-
	Total knee replacement	4 (18.2%)	-	-	-	-
	Time to TKR (years)	-	8.77	5.58	1.69	15.01
	Deep infection	0 (0%)	-	-	-	-
	HHS knee score	-	84.23	10.34	66	100
	Rasmussen score	-	13.27	3.47	6	18
	SF12—Physical	-	35.05	10.41	20.16	53.99
	SF12—Mental	-	53.03	10.55	29.35	65.73
HHS Knee Score	Excellent	15 (68.2%)	-	-	-	-
	Good	4 (18.2%)	-	-	-	-
	Fair	3 (13.6%)	-	-	-	-
	Poor	0 (0%)	-	-	-	-
Rasmussen Radiological Score	Excellent	3 (13.6%)	-	-	-	-
	Good	15 (68.2%)	-	-	-	-
	Fair	4 (18.2%)	-	-	-	-
	Poor	0 (0%)	-	-	-	-

Abbreviations: M: Male; F: female; R: right; L: left; AO: Arbeitsgemeinschaft für Osteosynthesefragen; OTA: Orthopaedic Trauma Association; G&A: Gustilo and Anderson; ExFix: external fixation; ROM: range of movement; TKR: total knee replacement; HHS: Hospital for Special Surgery.

**Table 2:** Pool of similar studies.

(A)								
Study	N	Sex (M/F)	Age (Years)	Open fx (%)	Time to Surg (Days)	Follow Up (Months)	ExFix Time (Weeks)	ROM (Degrees)
Ali (2003) [13]	20	8/12 (40.0%)	57.3	5 (25%)	-	30.0	19.1	115.5
El Barbary (2005) [14]	30	26/3 (89.7%)	41.4	9 (30%)	-	27.0	16.3	112.5
Ali (2013) [15]	25	16/9 (64.0%)	36.0	25 (100%)	3	30.0	14.0	112.0
Ahearn (2014) [4]	21	-	-	4 (19%)	-	31.0	-	-
Berven (2018) [30]	62	30/32 (48.4%)	55.7	7 (11%)	-	-	-	107.5
Bove (2018) [18]	14	13/1 (92.9%)	51.0	8 (57%)	19	-	22.0	-
Debnath (2018) [16]	15	13/2 (86.7%)	36.0	9 (60%)	5.6	19.4	14.6	110.0
Larsen (2019) [17]	22	14/8 (63.6%)	42.8	3 (14%)	-	112.8	24.1	-
Average	26.1	69.4%	45.7	39.5%	9.2	41.7	18.4	111.5
Ros (2022) [52]	22	11/11 (50.0%)	60.1	3 (13.6%)	10.4	74.0	24.1	111.4
*p*-values	0.8655	-	0.022	-	1	0.058	0.059	1.000
(**B**)								
**Study**	**Non-Union**	**Delayed** **Union**	**Pin Infection**	**Deep** **Infection**	**Osteoarthritis (%)**	**Good and Excellent Radiological Results**	**Good and Excellent Clinical Results**	
Ali (2003) [13]	-	-	7 (35%)	0 (0%)	17 (85%)	17 (85%)	16 (80%)	
El Barbary (2005) [14]	1 (3.3%)	3 (10%)	-	-	-	28 (93.3%)	25 (83.3%)	
Ali (2013) [15]	0 (0%)	0 (0%)	5 (20%)	1 (4%)	-	-	20 (80%)	
Ahearn (2014) [4]	0 (0%)	-	6 (28.6%)	0 (0%)	-	-	11 (52.4%)	
Berven (2018) [30]	3 (4.8%)	-	25 (40.3%)	6 (9.7%)	42 (68%)	-	-	
Bove (2018) [18]	1 (7.1%)	-	-	-	-	-	13 (92.9%)	
Debnath (2018) [16]	0 (0%)	2 (13.3%)	2 (13.3%)	0 (0%)	0 (0%)	10 (6.7%)	14 (93.3%)	
Larsen (2019) [17]	-	-	5 (22.7%)	0 (0%)	16 (73%)	-	-	
Average	0.8 (2.6%)	1.7 (7.8%)	8.3 (26.7%)	1.2 (2.3%)	56.4%	77.8%	80.3%	
Ros (2022) [52]	0 (0%)	4 (18.2%)	4 (18.2%)	0 (0%)	11 (50%)	17 (77.3%)	19 (86.4%)	
*p*-values	-	-	-	-	-	-	-	

Abbreviations: fx: Fractures; ExFix: external fixation; ROM: range of movement.

## Data Availability

The data presented in this study are available on request from the corresponding author. The data are not publicly available due to privacy concerns.
